# PVT1 Signaling Is a Mediator of Cancer Progression

**DOI:** 10.3389/fonc.2019.00502

**Published:** 2019-06-12

**Authors:** Camille Derderian, Akintunde T. Orunmuyi, E. Oluwabunmi Olapade-Olaopa, Olorunseun O. Ogunwobi

**Affiliations:** ^1^Department of Biological Sciences, Hunter College of The City University of New York, New York, NY, United States; ^2^Department of Radiation Oncology, College of Medicine, University of Ibadan, Ibadan, Nigeria; ^3^Department of Surgery, College of Medicine, University of Ibadan, Ibadan, Nigeria; ^4^Hunter College Center for Cancer Health Disparities Research, Hunter College of The City University of New York, New York, NY, United States

**Keywords:** PVT1, lncRNA, cancer, miRNA signaling, tumorigenesis, biomarker, 8q24

## Abstract

There is increasing evidence that PVT1 has oncogenic properties and regulates proliferation and growth of many cancers. Themolecular mechanisms of action of PVT1 are mediated, in part, by microRNAs (miRNAs). However, some well-established transcription factors involved in cancer cell proliferation share a common thread of microRNA associations with PVT1. Furthermore, these microRNAs are also involved in mechanisms that lead to the development of drug resistance in cancer cells. While several microRNAs have been implicated directly in PVT1-mediated tumorigenesis, significant steps need to be taken to elucidate these important relationships. We synthesize the current knowledge of the miRNAs and associated genes by which PVT1 contributes to tumorigenesis. Overall, the trend suggests a negative correlation of microRNA expression with PVT1. It is clear that future studies involving PVT1 should be carried out in conjunction with microRNA analysis and should include large scale lncRNA-miRNA-mRNA network analysis. Likewise, the relationship between established transcription factors such as *p53* and *MYC*, and processes like epithelial-mesenchymal transition may offer valuable insight into the yet unknown mechanisms of PVTI-mediated cancer progression via microRNA-dependent signaling networks.

## Introduction

Cancer is the second most common cause of death in the United States of America and a leading cause of morbidity globally (1). Global projections indicate an increase in new cancer cases per annum from 14.1 million in 2012 to 21.6 million by 2030. Although the rate of cancer related deaths are declining in the US, the rising costs of healthcare and the need to further improve cancer survival makes it a prevalent public health issue (2). In the US and elsewhere, there are disparities in risk factor exposure, access to screening, early detection, timely, and appropriate treatment as well-other determinants of poorer outcomes for cancer. Whereas, human behavior and societal factors play larger roles in cancer control, early clinical intervention, and greater success at understanding cancer biology remain important approaches for clinical impact. Thus, unraveling the mechanisms of cancer development and progression are critical for discovery of early detection tools and discovery of potential targets for treatment of cancer.

To further our understanding of the mechanisms and processes involved in the growth and progression of tumors, attention has been turned to long non-coding RNAs (lncRNAs). LncRNAs are ribonucleic acids (RNAs) that are longer than 200 base pairs, and do not encode proteins. Though previously discounted as useless regions of the genome, long non-coding RNAs are now recognized as important participants in epigenetic regulation, regulation of gene transcription, and other essential biological processes that are implicated in tumorigenesis. Many studies have highlighted novel roles of lncRNAs, but the exact mechanisms of action and causal relationships relating to their molecular actions are still largely unclear (3). The biogenesis of lncRNAs shares similar characteristics with that of mRNAs; RNA polymerase II is recruited to transcribe a section of DNA, and both undergo 5′ capping, splicing, and polyadenylation (4–7). The search for functional lncRNAs has been aided by this common process. Characteristic lysine trimethylation on histone 3 at the site of the promoter as well as a downstream site shows that RNA polymerase is being recruited to specific areas of the intron to create RNA transcripts (4, 7). Despite sharing some key similarities, lncRNAs diverge from mRNAs in important ways. They are generally shorter than mRNAs, are expressed at lower levels, and their primary sequences are not highly conserved. In addition, expression profiles for lncRNAs are specific across cell and tissue types (7–9). Key to understanding the biogenesis and function of lncRNAs in cancer is to elucidate the relationship between lncRNAs and miRNAs. Although the specific functions of all miRNAs remains unknown, they are generally thought to play an important part in the post transcriptional modification of genes (10). This makes them of specific interest to the study of pathways relating to lncRNA, as their interaction with these transcripts could influence gene expression in cancer.

The emergence of large scale comprehensive analysis of lncRNA-miRNA-mRNA networks is an important step toward understanding where lncRNAs fit into the biological processes behind cancer progression. Within the last year, these analyses have been done to further our understanding of diseases including chronic obstructive pulmonary disease, abdominal aortic aneurism, asthma, and cancer (11–14). By analyzing the differential expression of lncRNAs, miRNAs, and mRNAs, identification of key pathways and mechanisms of disease may become much clearer. Taking a wide approach such as this may provide key insight into aspects of the genome, like lncRNAs, that are not as widely characterized.

In humans, transcription of lncRNAs from human chromosome 8q24 may play a significant role in several types of cancer. The 8q24 chromosomal region is known as a gene desert, because of its lack of protein-coding genes. It contains a large number of risk alleles that are implicated in cancer, including the lncRNA Plasmacytoma Variant Translocation 1 (PVT1) as well as an associated protein-coding gene *MYC* (15). Mutations in this region such as copy number aberrations and single nucleotide polymorphisms (SNPs) have been implicated in the susceptibility to and progression of cancer (15). Bioinformatics analyses have identified PVT1 as one of a few key functional lncRNAs in many cancer types (16, 17). PVT1 is made up of 1,957 base pairs and has 9 to 12 exons (18). The exact biogenesis of lncRNA PVT1 is not characterized, but its association with factors like cell differentiation, metastatic disease, overall survival, and tumor stage, among other characteristics, has been widely studied (19, 20). In addition, lncRNAs are able to take many forms, including circular RNAs (circRNAs), which have recently gained recognition for their tissue- and stage-specific expression (7). CircRNAs are formed by back-splicing exons into a circular form and are very stable and not susceptible to degradation once formed. The recent characterization of PVT1 CircRNA shows promise for future discovery of biomarkers and drug targets for cancer control (17, 21, 22).

Due to differential expression across cell and tissue types, the mechanisms of action of PVT1 are largely unknown and likely cell and tissue specific (7–9). The complex interactions of PVT1, its proximity to the MYC oncogene, and its upregulation in cancer has made PVT1 a gene of significant interest for both screening and therapeutic targeting of cancer (23). While PVT1 is known to be associated with differential expression of some well-known genes, including MYC, it is important to also turn attention to other aspects of its function. Due to the ubiquitous nature of PVT1 upregulation in cancer, there is a need to synthesize the research on its potential utility within the cancer control continuum. Here, we describe the strong relationship between PVT1 overexpression and cancer progression, and then we discuss the expanding frontiers of PVT1 activity and its potential role in a lncRNA-miRNA-mRNA axis. We believe that PVT1 plays a pivotal role in compeititvely inhibiting miRNA and provide information for the formulation of hypothesis-driven research into PVT1 and evaluation of its functional mechanisms in tumorigenesis.

## Biological Significance of PVT1

### PVT1 Is Upregulated in Numerous Cancer Types

Numerous studies have evaluated PVT1 and circPVT1 function in tumorigenesis. Consistently, PVT1 is overexpressed in cancer cells compared to normal non-tumorigenic epithelial cells. It is found to be upregulated in bladder (24), breast (25–27), cervical (28), colon (29), colorectal (30, 31), gastric (32–35), lung (36–41), pancreatic (42), and prostate cancer (43–46), as well as clear cell renal cell carcinoma (18), esophageal squamous cell carcinoma (SCC) (20, 47), glioma (48–50), head and neck SCC, hepatocellular carcinoma (51–55), laryngeal SCC (56), malignant pleural mesothelioma (57), osteosarcoma (58–60), and papillary thyroid carcinoma (61), melanoma (62). Further, data from The Cancer Genome Atlas (TCGA) and independent analysis of human clinical tissue samples showed elevated PVT1 expression in cancer tissues in comparison to adjacent normal tissue or matched normal tissue (27, 31, 32, 45, 50). Analysis of circPVT1 expression in osteosarcoma, SCC of the head and neck, and gastric cancer also showed significant overexpression of circPVT1 (21, 22, 35). Differentiation between circular and linear PVT1 is possible with RNA sequencing, because circRNA does not map to the human reference genome with RNA-seq fastq (35). The upregulation of circPVT1 is a promising biomarker for cancer, because circular RNA is more stable and less often degraded than linear RNA (22). It is well established that PVT1 overexpression is widespread across multiple cancer types, making the lncRNA PVT1 of particular interest in the search for processes and pathways involved in cancer progression.

### PVT1 Expression Correlates With Various Clinicopathological Characteristics

Clinical studies evaluating PVT1 demonstrate that PVT1 overexpression is frequently associated with advanced disease and poor prognosis. Increased PVT1 expression is strongly correlated with shorter overall survival in RCC and colorectal carcinoma (18, 31). Increased circPVT1 expression was also correlated with shorter overall survival in osteosarcoma, head and neck SCC and gastric cancer (21, 22, 63). However, in gastric cancer, it was reported that overall and disease-free survival was highest with increased circPVT1 expression and decreased PVT1 expression, and lowest with the reverse (35).

While overall survival was negatively correlated with PVT1 expression in all the reported studies, other clinicopathological characteristics are not as consistent. For instance, in osteosarcoma and colorectal cancer, high PVT1 expression correlated with lymph node metastasis, but this correlation was not observed in ccRCC (18, 22, 31). Furthermore, in gastric cancer, there is a lack of consensus between increased PVT1 expression and lymph node metastasis (50, 62). Notably, advanced TNM stage significantly correlated with increased PVT1 expression in ccRCC and gastric cancer (18, 50). Conversely, PVT1 levels showed no correlation with estrogen or progesterone receptor positivity in breast cancer (27). Such variation across cancer types underscores the importance of clearly understanding the specific roles of PVT1 overexpression; differences in the effects of PVT1 overexpression in cancer can be seen at the cellular level as well.

## PVT1 Functions as a Driver of Tumorigenesis *in vitro* and *in vivo*

### PVT1 Promotes *in vitro* Viability and Proliferation

PVT1 significantly promotes cell replication, proliferation, and growth in cancer (18, 27, 31, 32, 45, 50). In evaluating PVT1's potential role as an oncogene and driver of tumorigenesis, there is evidence for its influence on cell viability and proliferation in various cell types. A knockdown of PVT1 via short interfering RNA (siPVT1) resulted in decreased cell viability in prostate cancer, melanoma, glioblastoma, gastric cancer, clear cell renal cell carcinoma, breast cancer, and colorectal cancer cell lines (18, 27, 31, 32, 35, 45, 50, 64). In glioblastoma cell lines, induction of apoptosis was significant in comparison to controls (50). Similar effects were observed in colorectal cancer, prostate cancer, and gastric cancer cell lines (31, 50, 64). The role of PVT1 in the cell cycle is demonstrated in studies showing that knockdown of PVT1 expression in gastric cancer cells, melanoma cells, and ccRCC significantly induced G0/1 arrest and reduction in S phase (18, 32, 62, 64). This suggests that the cells were not moving into the DNA replication phase and could not continue to proliferate. Since the loss of PVT1 expression consistently induced apoptosis and inhibits DNA replication, it is likely that PVT1 regulates the survival, growth, and division of cancer cells. Similarly, knocking down circPVT1 expression in gastric cancer cells decreased cell viability and reduced colony formation (32). However, evidence supporting circPVT1's regulation of cancer cell viability is still limited, and requires further research to make more robust conclusions on the matter.

### PVT1 Promotes *in vitro* Cell Migration and Invasion

Metastasis is the most common cause of cancer-related deaths (65). Tumor invasion and migration are critical components of the complex multi-step metastatic process. Although evidence supporting PVT1 regulation of cell viability and proliferation is solid, evidence for the influence of PVT1 on cell migration and invasion requires additional clarification. Numerous studies utilized transwell assays to evaluate the migratory and invasive effects of PVT1 knockdown or overexpression. In all cases to date, knocking down PVT1 decreased migration and invasion, while overexpressing PVT1 increased migration and invasion (18, 31, 32, 50). Notably, a diverse array of processes were implicated in PVT1 regulation of migration and invasion, including epithelial–to–mesenchymal transition, transcription factor activation, gene activation, and tumor suppressor inactivation.

### PVT1 Promotes Tumorigenesis in Mouse Xenograft Models

*In vivo* studies using mouse models have contributed to our understanding of the effects of PVT1 in human cancer. Mice injected with clear cell renal cell carcinoma, colorectal cancer, or glioma cells overexpressing PVT1 displayed significantly larger tumor volume than control cells, while those injected with cells with knockdown of PVT1 displayed significantly smaller tumor volume (18, 22, 32). Knocking down PVT1 significantly reduced tumor volume in bladder, prostate, breast, and lung cancer, as well as hepatocellular carcinoma and glioma (24, 25, 37, 48, 55, 63, 66). Overall, mouse xenograft studies confirmed that PVT1 overexpression increased tumor volume and decreased overall survival while knocking down PVT1 had the opposite effect.

### PVT1 Mediates Drug Resistance

Drug resistance (DR), and multidrug resistance (MDR), are major obstacles to cancer treatment. Patients who exhibit drug resistance experience poorer prognosis and lower overall survival. DR occurs when treatment becomes ineffective because the cancer has lost responsiveness to the agent being used (67). MDR occurs when the cancer becomes resistant to the pharmacological agent being used along with other treatments that are dissimilar (68).

PVT1 has been implicated in DR and MDR in many cancer types, including gastric cancer, colorectal cancer, and osteosarcoma. PVT1 expression was significantly elevated in drug-resistant cell lines in gastric cancer, colorectal cancer, and osteosarcoma compared to non-resistant cell lines, and all three cancer types expressed higher levels of PVT1 compared to matched non-cancerous tissues (21, 22, 64). Knocking down PVT1 in gastric cancer and osteosarcoma significantly increased cisplatin sensitivity and reversed drug resistance to doxorubicin and cisplatin in resistant cell lines (22, 32). Upregulating PVT1 significantly decreased cisplatin resistance in colorectal cancer (31).

In osteosarcoma, the *ABCB1* gene was overexpressed in drug-resistant cell lines, but reduced in resistant cell lines where PVT1 has been knocked down (22). *ABCB1* encodes for a pump that drives drugs out of the cell. It is implicated in multidrug resistance, because it is not specific to one type of cancer drug (69). The *ABCB1* gene mediates known DR associated molecules MDR1 and MRP1 (70). Both molecules were analyzed in colorectal and gastric cancers to see how they change with PVT1 knockdown and overexpression. Interestingly, mRNA levels of these molecules decrease with PVT1 knockdown and increase with PVT1 overexpression (31, 64) PVT1 is implicated in DR and MDR in many cancer types, but further study is required to fully understand the pathways that mediate this PVT1-related cancer drug-resistance.

## PVT1 Acts as a ceRNA to Interfere With miRNA Expression

The lncRNA PVT1 has been found to influence the expression of a diverse range of miRNAs in many cancer types. There are many networks that involve a cascade of interactions between lncRNA, miRNA, and mRNA in various diseases, including cancer (11–14). The influence of onco-miRNAs (promoting tumorigenesis) and suppressing miRNAs (inhibiting tumorigenesis) present evidence for targeted roles of PVT1. The nature of the interaction between miRNAs and PVT1, the correlation between miRNA expression and PVT1 overexpression, and the genes implicated in these interactions are summarized on Table 1. In some cancers, PVT1 is thought to act as a miRNA sponge. In this role, PVT1 acts as a competing endogenous RNA (ceRNA) by competitively binding miRNAs, therefore interrupting their regulatory functions. When PVT1 is acting as a sponge for a specific miRNA, knockdown of PVT1 will cause an increase in the miRNA expression. Conversely, the overexpression of PVT1 reduces miRNA expression (Table 1). Further study into the negative relationship between miRNAs and PVT1 expression will likely elucidate larger networks that involve genes that are known to be essential in tumorigenesis. For example, concomitant overexpression of PVT1 and upregulation of HIF-1a has been shown in hypoxic non-small cell lung cancer (39). HIF-1a is a known transcription factor that plays an important role in the adaptation to hypoxic environments, tumor angiogenesis, and apoptosis (69). In NSCLC, dual reporter luciferase assays confirmed that there was a direct sponging relationship between PVT1 and miRNA-199a-5p that correlated with an increase in HIF1a expression (17). The same trend was observed in gastric cancer with PVT1 and miRNA-186 (32). In clear cell renal cell carcinoma as well as breast cancer, PVT1 was found to competitively bind miRNAs from the miR-200 family.The miR-200 family has been shown to prevent tumorigenesis and malignancy (18, 26) miR-200 family expression significantly reduces epithelial-mesenchymal transition in mutant p53 expressing cells when exposed to a carcinogen (74). This suggests a link between the competitive binding of miR-200s by PVT1 with the loss of the protective effect of miR-200 expression on mutant p53 expressing cells. In glioblastoma, advanced tumor grade correlates with high expression of both PVT1 and the *MEF2C* gene. Both miR-190a-5p and miR-488-3p directly target the downstream MEF2C gene, keeping it from binding the JAGGED1 promoter, which has been implicated as a driver of glioma malignancy (50). The *MEF2C* gene also influences neurogenesis and has been implicated in other cancers (69). PVT1 can also cause drug resistance via miRNA sponging. In osteosarcoma, the induction of the PI3K/AKT pathway was found to be induced by PVT1 expression. This activation was found to contribute to gemcitabine resistance in osteosarcoma cells, and the upregulation of miR-152 increased cancer cell susceptibility to the drug (59). In pancreatic cancer, miR-1207-5p/3p inhibition, correlated with PVT1 overexpression, was associated with gemcitabine resistance. Overexpression of the miRNA increased drug susceptibility by targeting the SRC proto-oncogene and ras homolog family member A (42). Overall, it appears that many PVT1-regulated miRNAs may have a protective effect against tumor growth and proliferation.

**Table 1:** miRNAs linked to PVT1 expression and their associated genes and proteins.

**miRNA**	**Cancer type**	**Direct interaction with PVT1**	**Correlation with PVT1 overexpression**	**Associated genes/proteins**	**Reference**
miR-125b	Non-small cell lung cancer	Yes	Negative	E2F2	(38)
miR-126	Lung cancer	Yes	Negative	SLC7A5	(36)
miR-128	Colorectal cancer	Not specified	Negative		(30)
miR-128-3p	Glioma	Yes	Negative	GREM1	(48)
miR-133a	Ovarian cancer	Yes	Negative		(71)
miR-146a	Prostate cancer	Not specified	Negative		(45)
miR-150	Hepatocellular carcinoma	Yes	Negative	HlG2	(53)
miR-152	Os teosarcoma	Not specified	Negative		(59)
miR-152	Gastric cancer	Yes	Negative	CD151FCF2	(33)
mir-186	Esophageal squamous cell carcinoma	Yes	Negative	PTIG1	(47)
miR-186	Prostate cancer	Yes	Negative	Twistl	(43)
miR-186	Glioma	Yes	Negative	Atg7Beclin 1	(49)
miR-186	Gastric Cancer	Yes	Negative	HlF1a	(32)
miR-186-5p	Hepatocellular carcinoma	Yes	Negative		(52)
miR-190a-5p	Glioma	Not specified	Negative	MEFZC JA GGED	(50)
miR-192-5p	Human umbilical cord epithelial cells (not cancer)	Not specified	Negative	p53 CDKN1A GDD45A MDM2	(72)
miR-195	Non-small cell lung cancer	Yes	Negative		(40)
miR-195	Osteosarcoma	Not specified	Negative	BCL2CCND1 FASN	(60)
mir-199a-5p	Non-small cell lung cancer	Yes	Negative	HlF1a	(46)
miR-199a-3p	Hepatocellular Carcinoma	Not specified	Negative	None	(17)
miR-199a-5p	Hepatocellular carcinoma	Not specified	Negative	None	(17)
miR-200 a and b	Non-small cell lung cancer	Yes	Negative	MMP9	(41)
miR-200 family	Breast cancer	Yes	Negative	CDH1	(26)
miR-203	Esophageal squamous cell carcinoma	Yes	Negative	LASP1	(20)
miR-214	Hepatocellular carcinoma	Yes	Negative	EZH2	(51)
miR-26b	Colon cancer	Yes	Negative		(29)
miR-29c-3p	Hepatocellular carcinoma	Not specified	Negative	None	(17)
miR-30a	Papillary thyroid Carcinoma	Yes	Negative	IGFIR	(61)
miR-365	Hepatocellular carcinoma	Yes	Negative	ATG3	(54)
miR-424	Cervical cancer	Yes	Negative		(28)
miR-424-5p	Hepatocellular carcinoma	Yes	Negative	INCEP	(17)
miR-488-3p	Glioma	Not specified	Negative	MEF2C JACGD	(50)
miR-497	Non-small cell lung cancer	Yes	Negative		(37)
miR-497	Osteosarcoma	Yes	Negative	HK2	(58)
miR-497-5p	Head and neck squamous cell carcinoma	Not specified	Negative	TP53	(21)
miR-519d-3p	Laryngeal squamous cell carcinoma	Yes	Negative		(56)
miR-1204	Yeast cells	Not specified	Negative		(73)
miR-1204	Malignant pleural mesothelioma	Not specified	Negative		(57)
miR-1204	Breast cancer	Not specified	Positive	VDR	(25)
miR-1204	Broad	Not specified	Negative	MYC	(23)
miR-1205	Malignant pleural mesothelioma	Not specified	Negative		(57)
miR-1205	Prostate cancer (castration resistant)	Not specified	Positive	ECLN3	(46)
miR-1206	Malignant pleural mesothelioma	Not specified	Negative		(57)
miR-1207	Pancreatic cancer	Yes	Negative		(42)
miR-1207-3p	Prostate cancer	Not specified	Negative	FNDCl, fibronectin, androgen Receptor	(44)
miR-1207-3p	Malignant pleural mesothelioma	Not specified	Negative		(57)
miR-1207-5p	Malignant pleural mesothelioma	Not specified	Negative		(57)
miR-1207-5p	Breast Cancer	Not specified	Negative	STAT6 CDKNlA CDKNlB	(27)
miR-1208	Malignant pleural mesothelioma	Not specified	Negative		(57)

## Discussion

Numerous studies have provided evidence that PVT1 overexpression is correlated with cancer growth and proliferation both *in vitro* and *in vivo*. It is clear from human tissue and mouse xenograft studies that overall survival and tumor size in many cancer types is related to PVT1 overexpression. Conversely, when PVT1 is knocked down, tumor cell viability decreases, and apoptosis increases. Therefore, PVT1 is very likely involved in cancer cell growth and survival in human cancer. However, few causal relationships have been elucidated in terms of PVT1's mechanisms of action. Similarly, no studies have established the age- and gender-specific associations with PVT1 despite descriptions in breast and prostate tumors.

The role of circPVT1 in cancer should not be overlooked. Due to the paucity of research studies, it is difficult to conclusively state the role of circPVT1 in cancer. However, it is well established that high circPVT1 expression correlates with better overall survival in some tumors. circPVT1 studies should be included in comprehensive PVT1 studies because it may have independent effects that modulate PVT1 expression. circPVT1 has also been observed to be degraded to a linear structure by miRNAs. Therefore, detailed study of the circular form of thePVT1 RNA is necessary (75). It is important to characterize circPVT1 on its own and also evaluate its association with expression of the *PVT1* gene.

Clinically, a large burden of cancer stems from its propensity to metastasize and resist treatment. As these are areas of great interest, extensive studies to evaluate the potential actions of PVT1 have been done. This review has characterized pathways that appear to involve PVT1 in the progression of cancer as well as the development of cancer drug resistance. Notably, pathways involved with genes such as *p53* and *MEF2C*, the HIF-1a transcription factor, and the mechanism of EMT. These factors are well-characterized in cancer, and their association with PVT1 has the potential to provide major insights into its role in tumorigenesis. Further elucidating the correlation of the well-known *p53* with PVT1 would be important for understanding the latter's specific role in cancer. Overall, it seems that PVT1 is a major part of many cascades involved in the dysregulation of cancer cell growth. The diversity of potential pathways that PVT1 acts on makes it interesting and challenging to pinpoint directions for future study. However, the common thread of miRNA involvement is something that could streamline future research.

Important to this review is the critical role that miRNAs plays in PVT1's tumorigenic and drug resistant mechanisms. Depending on cancer type, different miRNAs appear important in the action of PVT1 in cancer. miRNAs have important regulatory functions that are modified when PVT1 is overexpressed. The sponging action of PVT1 reduces the number of miRNAs that are able to influence cellular processes, and therefore cancer is able to progress. In addition, specific miRNA analogs combined with reduction of PVT1 expression are able to even further reduce tumor growth, suggesting that miRNAs may collaborate with PVT1 to regulate tumor progression. These processes and, cause and effect relationships, are yet to be fully fleshed out. However, this is an important area on which future PVT1 research should focus.

Future research surrounding the elucidation of lncRNA-miRNA-mRNA networks is key to finding effective therapeutic targets in many cancer types. The widespread role of PVT1 and its function as a ceRNA make it an attractive candidate upon which to center future studies. Further research into the function of PVT1-regulated miRNAs in the protection of cells against tumorigenesis is an important step toward understanding how PVT1 serves to eliminate the cross-talk between miRNAs and their target genes. Ultimately, PVT1 is a promising lncRNA that holds promise for potential applications in diverse aspects of cancer healthcare delivery ranging from detection to treatment. It should likely be studied in conjunction with miRNA expression and action because they appear to be highly correlated in many cases. Associations with the protein-coding gene *MYC, p53*, and epigenetic markers should establish the programming actions of PVT1 in tumorigenesis. Processes such as EMT appear to be modulated by the expression of miRNAs and PVT1 and should be included in future studies involving PVT1.

## Author Contributions

OO and CD conceived of the article. CD, OO, AO, and EO-O gave intellectual input. CD, OO, and AO wrote the article. All authors approved of the article.

### Conflict of Interest Statement

OO is a co-founder of NucleoBio, Inc., a City University of New York start-up biotechnology company interested in developing PVT1-related molecular diagnostics and therapeutics. The remaining authors declare that the research was conducted in the absence of any commercial or financial relationships that could be construed as a potential conflict of interest.
